# Perceived barriers and opportunities for implementing an integrated psychological intervention for depression in adolescents living with HIV in Tanzania

**DOI:** 10.1186/s12913-024-11118-5

**Published:** 2024-05-28

**Authors:** Tasiana Njau, Dorkasi L. Mwakawanga, Bruno Sunguya, Agape Minja, Sylvia Kaaya, Abebaw Fekadu

**Affiliations:** 1 Department of Psychiatry and Mental Health, Muhimbili University of Health and Allied Sciences, Dar es Salaam Tanzania, United Nations Road, Dar es Salaam, P.O Box 65001 Tanzania; 2 Department of Community Health Nursing, Muhimbili University of Health and Allied Sciences, Dar es Salaam, Tanzania; 3 Department of Community Health, Muhimbili University of Health and Allied Sciences, Dar es Salaam, Tanzania; 4 Centre for Innovative Drug Development and Therapeutic Trials for Africa (CDT-Africa), Addis Ababa University, Addis Ababa, Ethiopia; 5 Department of Psychiatry, School of Medicine, College of Health Sciences, Addis Ababa University, Addis Ababa, Ethiopia; 6 Department of Global Health & Infection, Brighton and Sussex Medical School, Brighton, UK

**Keywords:** Adolescents, Mental health, HIV/AIDS, Depression, Integrated care, Psychological intervention, Implementation

## Abstract

**Background:**

Adolescents living with Human Immunodeficiency Virus (HIV) have an increased risk of depression, negatively affecting their adherence to antiretroviral therapy (ART) and treatment outcomes. Integrating mental health care in HIV care and treatment settings improves comprehensive care. However, integration remains challenging in Tanzania, like in other high-burden and low-resource settings. The overall objective of this work is to inform the development of a psychological intervention for depression in adolescents living with HIV (ALWHIV). We describe perceived barriers and opportunities for implementing an integrated, evidence-based psychological intervention to manage adolescent depression in HIV care and treatment centers (HIV-CTC) from the perspectives of adolescents, caregivers, and healthcare providers (HCPs) in Dar es Salaam, Tanzania.

**Methods:**

To inform intervention development and implementation, this study utilized a qualitative design through a phenomenological approach informed by the Consolidated Framework for Implementation Research (CFIR) to explore implementation barriers and facilitators in ALWHIV, HCPs, and caregivers. Forty-five in-depth interviews were conducted in three HIV-CTCs in Kinondoni Dar es Salaam. Audio records were transcribed verbatim and analyzed deductively through NVIVO software.

**Results:**

Barriers to implementing an integrated psychological intervention to address depression in ALWHIV included (A) poor mental health awareness among caregivers, adolescents, HCPs, and policy-makers, (B) high level of stigma against mental health care, (C) poor communication between adolescents and HCPs concerning mental health care, (D) lack of contextualized intervention of proven effectiveness and guidelines of mental health care, and (E) inadequate mental health care supportive supervision and mentorship. Facilitators for implementation included supportive infrastructure, positive pressure from HIV implementing partners, tension for change, and participant’s perception of the advantage of a psychological intervention as compared to just usual HIV care and treatment counseling.

**Conclusion:**

Despite several modifiable barriers to implementing a psychological intervention in HIV CTC, there were encouraging facilitators and opportunities for implementing an integrated, evidence-based psychological intervention to address depression in ALWHIV in Kinondoni Dar es Salaam, Tanzania.

**Supplementary Information:**

The online version contains supplementary material available at 10.1186/s12913-024-11118-5.

## Background

Adolescence comes with many cognitive, social, psychological, reproductive, and behavioral changes and transition issues [1]. It is also during this phase of life that many mental health challenges begin [2]. Mental health problems affect one in every five adolescents worldwide, making them the largest health problem experienced by young people globally [3, 4]. These problems can be exacerbated by stigmatized comorbidities. Adolescents living with HIV (ALWHIV) are reported to have greater psychological problems than those not infected by HIV [5]. In Tanzania, a study by Ramaiya and colleagues showed that ALWHIV experience high levels of stigma, difficulties in accepting and coping with HIV status, complicated grief following the loss of parents in the HIV pandemic, and violence—emotional, sexual, and physical—arising from their HIV status and orphan-hood [6]. These factors are reported to increase their vulnerability to depression, suicide and substance abuse [7, 8]. Depression is particularly common in ALWHIV and has been associated with impairment of developmental, leading to a wide range of negative mental, physical, and psychological outcomes as well as poor quality of life. In a recent meta-analysis, more than a quarter (26.07%) of ALWHIV had depression. Depression was highest among female adolescents (32.15%) and older adolescents (37.09%) [9].

In Tanzania, prevalence rates of depression in ALWHIV are reported to range from 12% (*N* = 182) to 47.1% (*N* = 209); this prevalence is three times greater than that of adolescents in the Tanzania general population [10–12]. The presence of depression in ALWHIV has been widely reported to affect antiretroviral therapy (ART) adherence [10], as well as increase morbidity, mortality, caregiver burden, and cost and decrease quality of life [7, 13–15]. When investigating the relationship between depression and ART medication adherence, Gonzalez JS et al. found via a meta-analysis that depression, even at a sub-clinical level, seriously reduced adherence to ART and that the effect was [16]. A substantial amount of the literature on ART adherence in Sub-Saharan African (SSA) countries, including Tanzania, has focused on factors that impact ART adherence in adolescents, and depression has been consistently reported across these studies to significantly affect adherence [16–21].

Despite the high prevalence of depression among ALWHIV in Tanzania, mental health services are often inadequate and not integrated into routine HIV care [21] leading to poor mental health outcomes and suboptimal medication adherence and care engagement [10]. Reducing depression in this age group has been reported to significantly improve ART adherence and, hence, clinical outcomes of this vulnerable population [22–25]. In Tanzania, while psychosocial interventions are provided, including counseling for adherence and psychological distress in adolescent HIV clinics, rarely are assessments made to identify and manage depressive symptoms in clinic-based standard operating procedures (SOPs). Integration of mental health services in Adolescent HIV care and treatment is a promising strategy to ensure adolescents receive treatment for depression at a place where they receive HIV medical care [26]. Within the Tanzania healthcare system, mental health integration in HIV care is an advocated strategy to increase accessibility to care, especially for ALHIV and is implied in the Tanzania Mental Health Policy [27]. Translation into practice has, however, been a challenge, and little is known in the Tanzanian cultural context about barriers and facilitators of accessing/providing services for recognizing and managing depression for adolescents in this resource-sparse setting.

Given the high burden of HIV and depression in ALWHIV in Tanzania, understanding barriers that limit the assessment, diagnosis, and management of depression for adolescents in HIV care and treatment centers (HIV-CTC) may inform implementation strategies for depression care and pave the way to addressing other common mental health disorders [28, 29]. The opinions of adolescents, caregivers, and healthcare providers are important in the design, development and implementation of HIV and mental health interventions within HIV CTCs [30, 31]. To address this evidence gap and explore the potential of implementing psychological health care targeting symptoms of depression and ART adherence in HIV CTCs, the current study was performed. This study aimed to explore perceived challenges and opportunities for implementing an integrated psychological intervention for managing depression in ALWHIV, with the ultimate goal to inform the development and implementation of a brief psychological intervention for depression in ALWHIV in Tanzania. To our knowledge, this study is the first to be conducted in Tanzania or sub-Saharan Africa on perceived barriers to formal mental health intervention implementation among ALWHIV.

## Methods

### Study design

We employed a qualitative study design through a phenomenological approach informed by the Consolidated Framework for Implementation Research CFIR [32] to explore the barriers and opportunities for implementing an integrated psychological intervention for depression in ALWHIV in Kinondoni district, Dar es Salaam, Tanzania. A qualitative approach was preferred to understand the subjective experiences of adolescents and their caregivers when accessing psychological care and the challenges HCPs face in providing such care in an integrated care setting [33]. We conducted 45 in-depth interviews among adolescents, caregivers, and HCPs in Kinondoni. In-depth interviews were found suitable for capturing opinions on implementation opportunities from adolescents and their caregivers as well as HCPs who are the primary stakeholders in the provision of adolescent care [34]. Selected CFIR constructs (supplementary file 1) were included to ensure that factors influencing intervention implementation are well incorporated.

### Study setting

This study was conducted in HIV Care and Treatment Centers (CTCs) of the Kinondoni Municipality in the Dar es Salaam region. According to the Management and Development for Health (MDH); a non-governmental organization that supports HIV care and treatment in Tanzania. Seven HIV-CTCs in Kinondoni provide care to the majority of ALWHIV. Three of the seven centers were purposefully selected with the help of MDH to the study sites because of their long experience running adolescent-friendly HIV care and treatment services and having experienced health providers.

### Study participants and recruitment

Forty-five [45] participants were recruited for this study, including 15 adolescents, 15 parents/caregivers, and 15 HCPs. We recruited participants from all three study sites using a purposive sampling technique based on their roles or experience with ALWHIV. HCPs included clinicians and nurse counselors delivering adolescent HIV care and treatment. HCPs were approached and recruited by TN (face-to-face), and selected based on their knowledge and experience (at least five years working with adolescents) about the dynamics of mental health problems in ALWHIV, the health system, and mental health services integration. Adolescents aged 11 to 24 were recruited from their respective clinics with the help of a familiar healthcare provider considering age, sex, awareness of their HIV status and their ability to communicate their experiences and give opinions in an articulate, expressive, and reflective manner [35]. Parents/guardians of ALWHIV were selected from the registry book of the individual center and invited to participate by phone based on their willingness to participate and having an adolescent attending services with emotional, behavioral, or adherence challenges. All interviews for all informant types were face-to-face and conducted at the clinic. Forty-seven potential participants were approached with two declining due to work-related reasons (one caregiver) and school reason (one ALWHIV).

We obtained ethical approval, institutional permission, and written informed consent/ assent from participants before the conduct of interviews. The recruitment was based on the saturation principle; when no new information was obtained from participants and redundancy was achieved [34, 36].

### Data collection process

We developed a semi-structured interview guide for HCPs, caregivers, and adolescents. The guides were initially developed in English and later translated into Kiswahili. The guides were developed based on a literature review [30, 37–43], informed by relevant CFIR constructs [32] and the researchers’ experience. The guides comprised open-ended questions and probes on barriers/opportunities to implementing integrated mental health care in HIV CTC for ALWHIV. The questions covered constructs of the CFIR, including intervention characteristics, outer setting, and inner setting. The thematic areas per participant type were as follows: For adolescents and their caregivers, we explored their experience and knowledge of depression, perception of the effect of depressive disorder on their lives, presenting problems, and barriers to care. We also explored the experience of modern and traditional methods of help, their understanding of the ideal treatment for depression, their opinion about implementing psychological interventions, and their advantages.

For HCPs, we explored their knowledge and experience working with adolescents with depression and their caregivers, including management and referral pathways, knowledge and experience of mental health care integration, awareness of evidence-based psychological interventions and their use for adolescent depression, challenges while integrating MH care for ALWHIV, and opportunities for implementing an integrated, evidence-based psychological intervention for adolescent depression. We also explored their views on what intervention components work and what valuable techniques are needed to provide psychological care successfully. TN and three trained female research assistants (RAs) with a degree in social work conducted the interviews. The RAs were selected based on their mental health care experiences and competencies in qualitative data collection. In addition, they received training on the study protocol, objectives, research ethics, data collection guides, and the research process.

All interviews were one-to-one and were conducted between May and June 2021. We conducted 15 in-depth interviews with each participant type (adolescents, HCPs, and caregivers). Before interviews, we obtained written informed consent from all study participants after explaining the purpose of the study, the risks and benefits of participation, and measures to keep shared information confidential. All participants also consented to the session being audio-recorded. All the interviews with adolescents and caregivers were conducted within respective CTCs in a private and quiet room chosen by the healthcare provider. HCPs were interviewed in their offices at each clinic. In addition, the lead researcher held meetings with HCPs to clarify information from the transcripts via phone calls/ZOOM meetings due to the COVID-19 situation in the country at the time of data collection and before developing the code book. All interviews were conducted in Swahili, a native language familiar to the researchers and participants. All interviews were audio-recorded and lasted between 30 and 45 min. For each interview, brief field notes supplemented the audio-recorded information during transcription. Interviews were conducted until saturation occurred for each group of participants (adolescents, caregivers, and HCPs) when no new information was obtained from participants and redundancy was achieved [34].

### Data analysis

Data analysis was consensus-based and iterative, following a deductive approach, using a predefined codebook with CFIR constructs (Supplementary material Table 1). A deductive approach in data analysis was preferred to ensure all psychological constructs relevant to intervention and implementation are considered [44]. Audio-recorded interviews were first transcribed verbatim. Next, thematic analysis [45] was conducted to identify key themes regarding perceived barriers and opportunities for integrating psychological intervention for depression in HIV CTC. TN, AM, and DM read and re-read the full transcripts to become familiar with the data and the context. TN then deductively developed a codebook based on the research objectives and predefined codes to guide the initial coding process. The codebook was imported into NVivo qualitative data analysis computer software to organize and manage data. Two researchers (TN and AM) coded five similar transcripts separately to validate the codebook. Then, they were compared for agreement on the final codes and their definitions. Other researchers (SK and DLM) independently reviewed codes and articles to improve the validity of the emerging themes. The analysis was done in Swahili to maintain the originality of the participants’ information, and descriptive quotations used to illustrate findings were translated into English.

## Results

### Demographic characteristics

Forty-five participants (15 adolescents, 15 HCPs, and 15 parents/caregivers) participated in this study. Of the 15 HCPs, 8 were clinicians, and 7 were nurse counselors. Eight [8] adolescents were female, seven [7] were male, and the mean age was 15.2 years. Most of the caregivers (nine of fifteen) had at least a primary level of education. Detailed social-demographic characteristics of participants are summarized in Table 1.

**Table 1:** Distribution of social demographic characteristics of the study participants recruited from HIV services for adolescents in Dar es Salaam, Tanzania

Participants	Adolescents *N* = 15	Care givers *N* = 15	Clinicians *N*= 8	Nurse counselors *N* = 7
Age range years	12–19	27–61	25–53	27–64
Mean age years	15.2	45.6	33	47.8
**Sex**				
Male	7	3	3	0
Female	8	12	5	7
**Education**				
None	-	1	-	-
Primary level	8	9	0	
Secondary level	7	5	0	2
Tertiary level	0	0	8	5

### Barriers to the implementation of psychological intervention

Barriers to implementing a brief psychological intervention to address depression and ART adherence in ALWHIV were related to the outer setting of the CFIR construct, namely, participants’ needs and resources. These barriers include inadequate mental health knowledge, negative mental health disorder attitude, stigma, communication challenges, availability and knowledge of evidence-based interventions, and pathways to care. Table 2 below summarizes these findings.

**Table 2:** Main themes and subthemes representing the main barriers and opportunities for implementing integrated psychological interventions at HIV-CTC in Dar es salaam

Themes	Sub themes
Inadequate mental health Knowledge	Limited awareness of own mental health problems
	Inadequate knowledge of available mental health services.
Negative mental disorders attitudes and stigma	Negative mental health attitude.
	Stigma to help seeking
	Stigma and referral
Communication	Understanding and response to mental health needs
	Challenges in establishing a working relationship
Inadequate knowledge and skills to manage depression	Lack of trained personnel
	Unclear referral system and pathway to care
	Poor knowledge of evidence-based treatment
	Lack of tools and guidelines for mental health intervention
Implementation opportunities	Favorable Implementation climate
	Relative advantage
	Need for the intervention

### Inadequate mental health knowledge

The inability to recognize symptoms of mental illness among the adolescents and caregivers was mentioned as a critical barrier to implementation, as it affects psychological help-seeking. Caregivers reported poor understanding of mental health-related symptoms. The challenge was partly due to somatic and behavioral presentation of depression in adolescents, as reported by this caregiver who works as a primary school teacher.

*“The problem was that she slept the whole day, crying and complaining about a headache and chest pain. Her father said it was laziness because her walking changed; she walked slower than usual. They could not figure out her problem at the clinic until doctors visited our school for mental health education on world mental health day. I finally realized it was depression.” (Caregiver, secondary education)*.

The role of HIV HCP in providing primary mental healthcare was not known. Even though available, most adolescents and their caregivers were unaware of the mental health unit within the primary health care hospital where HIV-CTC is located. They, however, were aware of HIV counseling services that are provided within HIV-CTC, including HIV medication adherence and sex education, as this parent/caregiver reported

*“There is no mental health unit here; I hear there is a hospital for crazy people in Dodoma but not in Dar es Salaam; there is no treatment for stress and depression…or maybe I do not know if they are treatable, I think there is no help perhaps until one is crazy. They (HCPs) only provide counseling on living with HIV, self-acceptance, and HIV medication usage” (Caregiver, primary education).*

### Negative mental health disorder attitude and stigma

A negative attitude towards mental disorders was also considered a barrier to implementing integrated mental health care. Participants believed that mental health professionals were there to care for people with severe incurable problems and that seeing a mental health professional would make a problem like depression chronic. It was also reported that mental illness symptoms are often associated with witchcraft. This healthcare provider has worked within HIV-CTC for nine years and describes his experience.

*Many people believe a person with a mental problem has been bewitched, so it is not even easy to say if that is depression because once you see persistent negative thoughts and behaviors, you cannot explain, plus the fact that our expertise to diagnose it is low, we end up losing them to witchdoctors.*

Adolescents commented explicitly on how mental illness is viewed negatively, and asking for help means one will be stigmatized for having a severe mental health problem.

*If you (an adolescent) get a referral for mental health care, your case is extreme, and doctors for crazy people will serve you. Adolescent, 15-year-old).*

Adolescents and their caregivers reported fear of double stigma due to the stigma toward mental illness within the community. In addition, seeking mental health care was thought to add another level of stigma (going Crazy) to the already existing HIV stigma. As a result, adolescents and caregivers were reluctant to accept referrals for mental health care, as described by this 18-year-old adolescent.

*I was afraid of everything and sometimes ran away because I wanted to be alone. My aunt suggested I see a doctor for crazy people. I thought, dah! So, I am crazy! I have HIV; and on the other hand, I am crazy … I did not go. You know it is only crazy people who go there (mental health care unit), which means I will be one of them.*

*Providers* reported a high stigma toward mentally unwell ALWHIV. This fear of double stigma was perceived to interfere with referrals to mental health care and hence a barrier to implementing a psychological intervention. A negative attitude about mental health was also observed in some providers, especially in how they address people with mental illness. This provider referred to it as ‘running mad,” as this provider with 6-year-experience in clinics narrates.

That is why others are lost to follow up…there is one we were following up on, and we realized she was completely crazy.

### Provider-adolescent communication challenges

Inadequate understanding or response to mental health needs was a key barrier to establishing an effective helping relationship, which may hinder the implementation of mental health interventions. Challenges in communication arose from the providers’ perceived busyness and limited time to listen to the concerns and needs of adolescents. Adolescents perceived HCPs as too busy with routine checkups and failed to listen to individual needs beyond managing HIV. They reported that HCPs did not adequately recognize or respond to their needs. As narrated by this adolescent, HCPs lack flexibility during service provision and could not support individual problems.

*When you go there[clinic], doctors are busy. Even if you describe your problems, they do not understand. It becomes difficult to ask for help. When you report a problem, they are usually busy with their issues making it difficult for them to understand the presented problem. You think of this, they think of that. An adolescent loses the confidence to go ask for help. (Adolescent, 17-year-old).*

Adolescents consistently perceive some providers as difficult to engage. For example, they perceive HCPs as prioritizing medication and not psychological support.

*Doctors do not understand the psychological burden you have. They think medication is the most important thing. We often have different priorities; you* (adolescent) *think of the emotional issues, but doctors think of medication. (Adolescent, 15-year-old).*

*On the other hand*, healthcare providers reported a lack of confidence in discussing mental health challenges with adolescents. Providers consistently expressed difficulties in approaching discussions about the emotional and suicide concerns with adolescents because they lack mental health training and hence unaware of what needs to be said or done. Some thought the mental health needs of the adolescents were less critical when compared to checking for viral load and CD4 count. They also thought addressing depression symptoms was less crucial than taking ART.

*There is a problem! We do not know because we are not trained. So, we only advise stopping overthinking and using medication (ART). First, you let them know that having HIV is not a death sentence, and then you go on with more important things like checking CD4, viral load, and filling their prescription. (Clinician, 5-year experience).*

### Lack of culturally acceptable intervention and intervention manual

None of the HCPs knew of any evidence-based psychological intervention to address depression. Providers reported having no formal training in identifying and managing depression, limiting their ability to help ALWHIV even when they want to.

*We do not deal with mental health problems. The biggest challenge is the lack of intervention skills and expertise to diagnose depression. Our clinic does not have a psychologist to ensure they get treatment. (Nurse counselor, 11-year-experience).*

Intervention and training manuals, screening tools, or procedures for psychological interventions were reported to be absent by all HIV-CTC, hindering implementation.

*Yes, we were not trained about mental health, but there are not even appropriate psychological intervention guidelines or a simple form that an adolescent can fill out that we can at least learn to use. We have manuals for TB, for example. So why not for depression while they* [government] *know how serious this problem [*depression*] is. (Clinician, 8-year-experience).*

### Pathway to care

The lack of a transparent referral system was perceived as a significant implementation challenge for HCPs in all centers. HCPs did not know where or when to refer an adolescent “with depression.” Even though there might have been a psychiatric nurse or a clinical officer trained in psychiatry within the hospital where the clinic is located, providers did not know that suicide attempts warranted referral to mental health care. Some believed to have not seen an adolescent with severe enough presentation to warrant referral but at the same time reported a case that ended with completed suicide. To these providers, only psychosis would be a reason for referral to specialized mental health care, as narrated by this provider.

*If we get a severe case requiring a psychiatrist, it will be challenging because we do not know how or where to refer such cases. Even though we have not yet seen a severe case, a clear understanding of the referral pathway is necessary. Perhaps we miss the diagnosis. For example, it was too late when one case ended up with complete suicide when we questioned whether the deceased was depressed. (Clinician, 5-year-experience).*

### Opportunities for implementation of psychological intervention

Opportunities for implementing an integrated psychological intervention for depression were evident and widespread across four constructs from two CFIR domains, namely inner setting (implementation climate, tension for change, positive pressure) and intervention characteristics (relative advantage).

#### Implementation climate

The main opportunity for implementing a brief psychological intervention was the availability of supportive health facilities infrastructure that may enable the integration of psychological interventions within HIV CTC. Youth clubs were reported as one of the possible venues to extend mental health education. In addition, HIV-CTC were reported to have well-structured counseling rooms, and at least two nurse counselors placed to provide psychosocial care; hence, they believe it is feasible to implement integrated psychological interventions within the existing facilities.

*The environment here is conducive to accommodating the provision of psychological service. The clubs are perfect areas to provide this education* (psychological health education). *We also have counseling rooms available and in use with two of us* (nurse counselors*), the right people to deliver the intervention for depression. (Nurse counselor, five-year experience).*

### Tension for change

The other opportunity for implementing a brief psychological intervention was the perception that the intervention would bring about access to knowledge and information about mental health problem assessment and treatment. To HCP, the intervention was thought to be helpful to adolescents and providers who have been burned out because of the inability to help adolescents with mental health problems. In addition, they think having the skills to address depression in adolescents will improve their confidence to effectively listen to issues presented to them by the adolescents, something that they currently avoid doing because of the guilt of not being able to help.

*Nothing is bad like seeing an adolescent struggling with a problem that he expects you to help, but you cannot. It is boring, and the work becomes tough and painful. As providers in the CTC unit, we urgently need mental health training. The knowledge will give us the confidence to listen and address adolescents’ mental health challenges. We currently distract them with a fear of being unable to help them, leading to a circle of self-blame, pain, and regret for not being helpful. (Nurse Counsellor, 14-year-experience).*

### Positive pressure

HCPs’ perceived pressure from HIV-implementing partners was a significant favorable influence because it “pushed HCPs to strive for excellence” in data registration, reporting, and service delivery.

*You know MDH [*a non-governmental organization that supports HIV service provision) *will be a reasonable force in making sure the implementation is successful. The good thing is that they are aware of the challenges adolescents and HCPs face, which is why there is that pressure to follow clinic SOPs. (health care provider, 5-year experience)*

### Relative advantage

Caregivers perceived the implementation of a brief psychological intervention to address depression would be advantageous when compared to using non-evidence methods to address depression.

*The treatment availability will take away a great burden from us. We will truly appreciate getting psychological services because we are suffering, not knowing what else we can do. I have exhausted all options. I started taking my daughter to traditional healers, pastors, and local counselors on the street, but nothing seemed to help. She will be okay for one day and returns to her world the next day. If there is a possibility of getting the service here, it will be helpful. (Caregiver, primary education).*

HCPs perceived that a brief psychological intervention would give them an advantage compared to routine activities and other routine interventions that address mental health problems through trial and error. Participants also indicated that supervision and on-the-job training would help them learn new competencies from psychologists or experienced fellow counselors that will help them standardize the delivery of psychological intervention hence improving the quality of mental health services within HIV-CTC. In addition, the availability of an intervention manual will help them become more conversant with intervention components and activities over time.

*The truth is that it will give us skills to improve what we have been doing. It will not be something new from what we have been doing, but rather enhancing our knowledge and skills to make us do it better. So, instead of doing trial-and-error game counseling, according to what we see is correct, we confidently do what is supposed to be done. Intensive training will, however, be required. (Nurse Counsellor, 7- years of experience).*

Consistently, adolescents thought the intervention would be helpful. Most of them felt that the services would benefit them and their peers whom they see struggling with depressive symptoms. In addition, they thought youth clubs would be an excellent venue for awareness.

*Yes, services should be available because many of us need them I am better now but will go if the service is available. We should be informed when they start. I know many adolescents will also go for the service. We should be announced because we are many, and we will crowd there because I feel everyone somehow has this problem. I will recommend the service”. (Adolescent, 19-year-old)*

## Discussion

In this qualitative exploration, we have highlighted barriers and opportunities for implementing an integrated psychological intervention for depression in HIV-CTC in Dar es Salaam, Tanzania. The Barriers described in this study are not only similar to those reported in other studies of mental health in HIV CTCs but also emphasize some unique issues and provide explanations that were specific to adolescents with HIV and add a vantage viewpoint of healthcare providers and caregivers.

In line with a study in Rwanda that reported poor mental health knowledge and a negative attitude towards care-seeking as a barrier to mental health care [46], participants in this study most frequently endorsed barriers related to a lack of knowledge about mental health and the available help, stigma, and negative attitudes towards mental disorders and help-seeking as a foreseen challenge to mental health care that may (if not well addressed) negatively affect implementation of psychological intervention for adolescents in HIV-CTCs. Improved mental health knowledge would help adolescents and their caregivers recognize signs of mental health problems like depression, which may encourage health-seeking behavior and intervention utilization.

Caregivers and adolescent participants perceived public stigma and embarrassment associated with mental health problems, negative expectations and attitudes toward mental health professionals, and help-seeking as a sign of the severity of the problem and one’s weakness. HIV-related stigma is often intertwined with other sources of stigma, including those associated with mental health. These findings indicate a need for strategies that improve mental health awareness and reduce stigma to be put in place before implementing mental health interventions within HIV care settings in Tanzania.

In this study, HCPs’ capacity to address depression in ALWHIV was compounded by a lack of knowledge and skills in depression assessment and management. None of the HCPs had ever heard of psychotherapy and were not aware of any evidence-based intervention for depression. This lack of awareness may be related to gaps in training /curriculum for Tanzania’s crucial primary healthcare workforce (clinical officers, assistant medical officers, and nurses). Addressing these barriers through programs that enhance service accessibility, and expand the role of CTC providers in providing mental health awareness should be prioritized.

The findings of this study add important information about the mismatch between the unique mental health concerns of ALWHIV and the failure of providers and systems to meet those needs. Broadly, participants cited consistently that poor mental health care was received due to the quality of interaction and communication difficulties with healthcare providers, lack of intervention, and mismatch of adolescents’ needs with the focus of care provided.

Participants in our study specifically described their experiences of having their psychological problems invalidated by providers. While HCPs prioritized prescribing ART, adolescent participants perceived psychological support was most needed. These Discrepancies in treatment priorities can negatively impact access to care and the feasibility of implementing an intervention to address depression in ALWHIV. This differing priority was partly accentuated by healthcare’s poor communication skills and incapacity to manage depression. HCPs’ communication and effective helping skills need to be improved to facilitate help-seeking, adherence, and of successful implementation of mental health intervention [18, 47]. Offering training, supervision, and continuous support for the HCP are also critical, supported by literature [27, 39, 48], and respond to the perceived need for high-quality training and supervision from appropriately experienced and qualified experts to support mental health services delivery [49]. In addition, providing an intervention manual that is clear and easy to follow and good quality supporting materials such as well-structured, highly manualized interventions will be more easily mastered by nurse counselors to ensure adherence to treatment procedures. The availability of training materials that fits healthcare providers’ training needs was also suggested by a study from a high-resourced setting as a factor contributing to the effective implementation of intervention [50].

The lack of knowledge about the mental health system and how and where to refer adolescents with mental health problems to HCPs in this study was striking. Adequate knowledge and understanding of mental disorders and available services, when and where to refer, can facilitate early recognition, appropriate help-seeking from the adolescent, and adolescent adherence to the intervention or services when provided [46].

On the other hand, the findings of this exploration indicate an absorptive capacity for implementing psychological intervention within HIV-CTC. A shared receptivity of psychological intervention among adolescents, caregivers, and HCPs suggests the possibility that the implementation of a psychological intervention will be rewarded, supported, and expected within the HIV-CTC. Fostering implementation of health services research findings into practice. This finding aligns with the recommendation from a consolidated framework for advancing the implementation science [32]. In line with the WHO Mental Health Gap Action Programme (mhGAP) [51], participants in this study recommend psychological intervention as the first-line treatment for depression in ALWHIV.

The presence of existing Counselling services and nurse counselors indicates a possible opportunity for integrating a brief psychological intervention within the current counseling services. There is growing evidence in LMIC for the acceptability, feasibility, and effectiveness of culturally appropriate psychological interventions delivered by well-trained and supervised non-mental health specialists [25, 52–56]. Task shifting [48] may be utilized in Tanzania to provide practical, culturally appropriate, and feasible psychosocial interventions for ALWHIV.

Caregivers’ perception that psychological intervention will improve adolescents’ health outcomes, and the adolescents’ willingness to receive the intervention suggests an opportunity for adaptation or development of psychological intervention and a potential for successful implementation of a culturally acceptable intervention within HIV CTC in Tanzania. Counselors’ willingness and perception that they are required to address depression within the scope of their work in HIV-CTC indicates how development and implementation of a psychological intervention will fit with existing HIV healthcare workflows and systems in Tanzania. HCPs’ perception of the current practice in addressing adolescent mental health as needing change indicates a tension for changing the practice that is not evidence-based or effective and a need for e effective, culturally appropriate psychological intervention to address depression in the HIV-CTC [32]. A successful development and implementation of a psychological intervention within HIV-CTC needs to consider CFIR constructs that promote key stakeholders’ receptivity to interventions, such as enhancing a positive tension for change and aligning the intervention with relative priorities as identified in this study. Formally engaging adolescents, caregivers, and HCPs as planning team members to inform the development and integration of psychological intervention for depression will address workflow and system complexity and ensure compatibility of the intervention with HIV-CTC culture, which is key in promoting high implementation fidelity [57]. Future research needs to develop and implement an evidence-based psychological intervention that is feasible, appropriate, and customized for Tanzania’s care setting.

### Strengths and limitations

This study is the first known to us that comprehensively explored barriers and facilitators for implementing a psychological intervention for depression in HIV-CTC in Tanzania. The use of CFIR constructs enhanced the ability of these findings to inform implementation strategies. Triangulation and a robust analysis approach reduced the likelihood of common information bias when obtaining self-reported data. Triangulation that was achieved by collecting data from HCPs, adolescents, and caregivers enhanced the credibility of these findings. We also used participants’ quotes in the results to support the researchers’ interpretations. Unfortunately, we did not conduct back translation to the interview guides and descriptive quotations, which may have made it difficult to spot potential differences in meaning. This limitation was minimized by conducting the analysis in Swahili to maintain the originality of the participants’ information, and only descriptive quotations used to illustrate findings were translated into English. The use of qualitative methods and purposeful sampling limit the generalizability of these findings to the larger population of ALWHIV; however, these results are hypothesis-generating. Finally, we used the Consolidated Criteria for Reporting Qualitative Studies (COREQ) checklist (*Supplementary Table*2) to ensure Quality reporting of this study’s findings [58].

## Conclusion

Perceived barriers and opportunities to implement an integrated psychological intervention for depression in ALWHIV in Dar es Salaam cut across factors that affect HCPs, adolescents, and caregivers. The barriers include the inability to recognize symptoms of depression, stigma, poor communication skills, inadequately trained HCPs, and lack of intervention. Implementation opportunities include strong support for psychological intervention delivered within HIV-CTC from adolescents, caregivers, and HCPs, a favorable implementation climate within HIV CTC, a positive tension for change, and participant’s perception of the advantage of implementing a psychological intervention versus usual HIV care and treatment counseling. The findings of this study suggest an urgent need for an intervention that will be and implemented to address depression specifically for ALWHIV, and that can be delivered in an integrated HIV care and treatment facilities in Dar es Salaam Tanzania and possibly similar settings in SSA.

### Electronic supplementary material

Below is the link to the electronic supplementary material.

Supplementary Material 1

Supplementary Material 2

Supplementary Material 3

## Data Availability

All data generated or analyzed during this study are included within the article.

## Abbreviations

ART: Antiretroviral Therapy
AL WHIV: Adolescents Living with HIV
CDT-Africa: Centre for Innovative Drug Development and Therapeutics Trial for Africa
CFIR: Consolidated Framework for Implementation Research
HCPs: Healthcare providers
HIV: Human Immunodeficiency Virus
HIV-CTC: HIV Care and Treatment Centers
IRB: Institutional Review Board
MDH: Management and Development for Health MDH
SOPs: Standard Operating Procedures
SSA: Sub–Saharan Africa
