# Supplementary material for: Perceived barriers and opportunities for implementing an integrated psychological intervention for depression in adolescents living with HIV in Tanzania
Source: BMC Health Serv Res. 2024 May 28;24:672. doi: 10.1186/s12913-024-11118-5 (PMC11134697; doi:10.1186/s12913-024-11118-5)
Supplement: Supplementary file 1 — Supplementary Material 1 [file 12913_2024_11118_MOESM1_ESM.docx]

| **Supplementary Table 1: CFIR constructs and subconstructs used to analyze study Perceived barriers and opportunities for implementing an integrated psychological intervention for depression in adolescents with HIV in Tanzania** | |
| --- | --- |
| **CFIR Domains** | **Constructs and Subconstructs Used in Final Analysis** |
| Outer setting | Participants’ needs and resources |
| Inner settings | Implementation climate |
|  | Tension for change |
|  | Positive pressure |
| Intervention characteristics | Relative advantage |
